# “And when will you install the new water pump?”: disconcerted reflections on how to be a ‘good’ Global Health scholar

**DOI:** 10.1186/s12992-023-00919-8

**Published:** 2023-03-21

**Authors:** Robert A.J. Borst, Rik Wehrens, Roland Bal

**Affiliations:** Erasmus School of Health Policy & Management, Erasmus University Rotterdam, P.O. Box 1738, Rotterdam, 3000DR the Netherlands

**Keywords:** Fieldwork, Research impact, Fair collaboration, Projectification, Postcolonial STS, Disconcertment

## Abstract

**Background:**

While critique on Global Health is not new, recent years show a surge of criticism on the field’s colonial legacy and practices specifically. Such accounts argue that despite Global Health’s strive for universality and equity in health, its activities regularly produce the opposite. The epistemic privileging of Northern academics and scientific method, further augmented by how Global Health funding is arranged, paints a picture of a fragmented field in which ‘doing good’ has become a normatively laden and controversial term. It is specifically this controversy that we seek to unpack in this paper: what does it take to be a ‘good’ Global Health scholar?

**Results:**

We used Helen Verran’s notion of ‘disconcertment’ to analyse three auto-ethnographic vignettes of Robert’s Global Health ‘fieldwork’. We illustrate that disconcertment, a bodily and personalised experience of unease and conflicting feelings, may serve as an important diagnostic of conflicting imperatives in Global Health. Robert’s fieldwork was entangled with incongruous imperatives which he constantly had to navigate through and that repeatedly produced disconcertment. The contribution that we seek to make here is that such disconcertment is not something to defuse or ignore, but to take seriously and *stay with* instead.

**Conclusion:**

Staying with the disconcertment serves as a starting point for conversations about ‘doing good’ in Global Health fieldwork and creates opportunity for making Global Health teaching and projects more reflexive. The paper thereby positions itself in discussions about fair collaborations between the Global North and South and our analysis offers a set of considerations that can be used by Northern scholars to critically reflect on their own role within Global Health.

## Background: practicing ‘good Global Health’

“Never forget to include (…) the Chinese proverb “Give a man a fish, and you feed him for a day, teach a man to fish and feed him for a lifetime.” How else will your readers know yours is a global health paper? It will also show that you have taken the time to understand local customs and have connected with the community on a deep level.”

[1]

Over the course of more than thirty years, actions dedicated to improving health globally have increasingly institutionalised. This institutionalisation of ‘global health’ happened to the extent that it is currently often referred to with a capitalised proper noun: Global Health. Global Health has shown to be difficult to demarcate, but its practices share their normative ambition for universality and equity in health: all people should have an equitable state of health and well-being [2–4]. This aspiration is reflected in the field’s interventionist nature, where most studies focus on improving the health of specific populations by applying novel interventions and measuring the successes thereof. On a more systemic level, Global Health actors are guided by the United Nations’ third Sustainable Development Goal, which calls for ensuring “*healthy lives and promote well-being for all at all ages.”* What this shows is that, in theory, Global Health aspires change and improvement whilst using agendas and goals to guide that process.

There are, however, increasing sentiments that Global Health does not live up to its claims, or even (re)produces the problems that it seeks to address [5–9]. Such sentiments focus on a misalignment between Global Health’s aspirations, claims, and achievements. Criticism on Global Health as such is not new [10], but recent years have shown a surge of critique specifically focussed on the field’s colonial legacy, its preoccupation with biomedical scientific knowledge, and the unbalanced nature of Global Health funding – predominantly coming from the ‘Global North’ [11–16].^1^

When looking at the themes around which criticism on Global Health revolves, it becomes clear that while the field aspires universality and equity, its practices often fail to live up to these aspirations. The privileging of the knowledge and methods of Northern scholars, for instance, may result in research that is not equipped for offering local solutions to improving health [5, 17]. Overall, these issues may best be summarised in terms of a conflict between how Global Health *ought to be done* and how *it is done in practice*. But that does not mean that this is merely a matter of wrongly acting on the right intentions. The challenge within Global Health is not to throw out the baby out with the bathwater, but to conclude that the water *is* spoiled nonetheless: while there have been significant improvements in health globally, these improvements do not justify the inequities and injustices that are *also* attributed to the field. One of the questions that this introduces is how Global Health scholars might consider and reshape their own role within an increasingly disputed field? This requires more personalised and reflexive accounts from Global Health scholars on what it means to ‘do good’ in practice. While such accounts by themselves will not have the thrust to overhaul how Global Health is funded and arranged, they can stir debate and cause Global Health researchers to make their own practices more reflexive.

In this paper, we will analyse auto-ethnographic vignettes of Robert, who is the first author of this paper. Robert is an early career researcher who has been active in Global Health for eight years now. He has experienced his work to be a constant struggle between common norms within Global Health and what he deemed to be ‘good’ Global Health scholarship. Working in Global Health excited him and he generally felt that he was doing meaningful work. Yet, there were also numerous occasions during his work in the Global South where he felt uncertain and uncomfortable. In his capacity as Northern Global Health scholar he often had doubt about how to productively position himself towards his Southern colleagues, or the topics of study.^2^ At the same time, Robert’s friends and colleagues back home saw his fieldwork as sensational, adventurous, and an endorsement of academic performance. The tensions described above where thus not something external to Robert as Global Health scholar, but he was very much complicit in them (cf. [18]). We will therefore reflect on what Robert experienced as conflicting situations in his Global Health work and what these conflicts say about practicing ‘good Global Health’.

We argue that the use of Robert’s auto-ethnographic vignettes is suitable and appropriate for several reasons. First, we argue that current contemplations over ‘what is wrong’^3^ with Global Health sometimes unsatisfactorily address the personal and affective reflections of Global Health scholars, both from the South and North. This is particularly salient given that such ‘soft signals’ can hint at more systemic issues within Global Health (cf. [19]). Second, by consistently focussing on systemic elements only, Global Health scholars defuse the uncertainty and unease in these experiences; consequently reverting to a logic of ‘blaming the system’. Instead, we want to embrace such reflections, deconstruct them, and show how they can be important instruments for changing Global Health from within. Third, we see the analysis of auto-ethnographic vignettes as a way to discuss issues within Global Health that are likely recognisable to other (early career) researchers, but which generally remain unsaid.

To be concrete, we foresee two contributions that this paper can make to Global Health literature and practice. First, we identify systemic features, or imperatives, within Global Health that prescribe what it means to be a ‘good’ Global Health scholar. We thereby also highlight the expectations that come with doing good. Second, we suggest that moments of disconcertment have an important signalling function: feeling of unease during fieldwork may, for instance, hint at a conflict between project objectives and local priorities. Paying more attention to such signals can cause Global Health scholars to interrogate their own position and role, but analyses of disconcertment can also inform teaching programmes and facilitate more reflexive project organisation. For example, if a project collaboration feels unfair, it is important to take these feelings seriously and explore how the project can be changed for the better. The analysis of personal reflections can thereby support the creation of a more reflexive Global Health in which other, diverse, logics and epistemic practices can be organised and valued. To make that contribution, we will first present the (auto)ethnographic approach that was used and describe the concepts that allowed us to deconstruct Robert’s struggles whilst working in Global Health.

## Methods and theory

In this paper, we use theory from the field of Science and Technology Studies (STS) to perform an analysis of auto-ethnographic vignettes. Our analysis builds on the work of Helen Verran, in particular her analyses of ‘disconcertment’ [20, 21]. Verran uses the term disconcertment to describe the experience of bodily ‘glitches’ when different ideas and values intersect [22]. These glitches are often irrepressible responses that happen when experiencing seemingly contradicting logics [23].

Verran famously described the concept of disconcertment in relation to practices of quantification in Nigerian classrooms [20]. Verran, at that time working as lecturer at the Nigerian Institute of Education, was meant to train educators in the use of numerical systems. In one of her essays, Verran narrates an observation of one of her students (Mr Ojo). Mr Ojo was training his pupils in the measurement of body length. Instead of using the standardised technique that Verran had taught Mr Ojo, he prepared and worked with a technique based on the Yoruba numerical system. Verran recalls her *“confused feelings of delight and suspicion, failure and success” (p. 140)* when noticing the triumph of Mr Ojo and his technique, despite its complete deviation from the intended lesson structure [22]. Such mixed feelings, and the irrepressible bodily responses that they can produce, is what Verran refers to as disconcertment.

Moments of disconcertment can have an important analytical value. Verran & Christie [21] describe that this value lies in *“being suddenly caused to doubt what you know”* (ibid., p. 53). The doubt that stems from disconcertment provides an opening for studying underlying dynamics and what alternative sorts of knowledge could play a role in the disconcerting moments. It is therefore important not to let disconcerting moments pass by, but to study and articulate what generated the disconcerting moment. By explicating such fundaments of disconcertment, as it were, we aim to account for the institutions, normativities, and practices which reproduce the misalignment between Global Health’s aspirations and achievements.

The methodological approach that we apply in this paper is that of writing-as-inquiry, which is common for qualitative auto-ethnographic studies [24]. In the case of our study, this means that we collectively and iteratively analysed and described moments of disconcertment that happened during Robert’s fieldwork, rather than performing the analysis prior to the actual writing. We increased the quality of this approach by using a strict paper trail [25, 26]. This paper trail involves fieldnotes, photos, e-mails, and diaries that covered eight years of fieldwork in different countries in the Global South. Occasionally, Robert narrated the context of disconcerting moments to Rik and Roland during the analysis. These narrations were written down as thick descriptions [27], which were used as an additional source for the auto-ethnographic vignettes.

We analysed all our data abductively [28]. Abductive analysis is an established mode of inquiry that allowed us to switch between generating new conceptual insights from the data and using existing (conceptual) literature as analytical framework. To be concrete, we first performed a round of open-coding which was sensitised by Verran’s notion of disconcertment. We then compared these codes to the contemporary critical Global Health literature (as cited in this paper). Based on this comparison, the three themes that would cover most data were: impact, collaboration, and project organisation. For each theme, we selected a moment of disconcertment in the data for which we found sufficiently rich data and where we could triangulate the account. Our final step was to collaboratively analyse these vignettes for normative expectations about what it means to be a good Global Health scholar and to describe them in relation to the literature in this manuscript.

For Robert, the analytical process presented itself as a *mise en abyme*: he experienced the description of these moments in itself as disconcerting and constantly sought to justify his words towards different accountability networks: how can Robert, for instance, do justice to his ‘fields’, the decolonisation of Global Health, his university, supervisors, colleagues, and his own values at the same time? This additional layer of disconcertment offers a unique opportunity for further reflection and analysis on why it is so uncomfortable and confronting to write about his own role in Global Health.

Following Verran [20], the results section of this paper follows a structure where we first present an auto-ethnographic vignette. The three vignettes in our results section each resemble one of the key themes of our analysis (i.e. impact, collaboration, and project organisation). Subsequently we discuss the parts within these vignettes that Robert experienced as disconcerting, including the different actors and elements that played a role in that moment. Finally, we discuss these deconstructed moments of disconcertment in relation to the (critical) Global Health literature.

## Results

*“—Yes, but uncle Deng, may I ask something?*

*My father, noting the man’s good manners, sat down and nodded.*

—*You didn’t tell us the answer: what is the what?*

*My father shrugged. —We don’t know. No one knows.”*

[29]

Robert has been active as a global health researcher for eight years now. In these years he travelled to numerous conferences across the world, visited ‘fields’ in various countries, and spent hours trying to make sense of what he measured, observed, and was told. Robert is now a frequent flyer and has a drawer at home packed with power adapters, foreign currencies, sim-cards, notebooks, and old conference badges. While working with numerous colleagues and friends from abroad has brought him tremendous joy, his experience in Global Health has also left him frustrated and somewhat estranged from his initial believes that he *is* contributing to better health, *globally*. This frustration arose at multiple moments throughout Robert’s work as a global health researcher. He experienced different conflicts between standard procedures in global health research and the realities he encountered in practice. In this article, we argue that these micro-level conflicts mimic wider tensions between epistemic practices within global health, and more systemic, normative aspects that are inscribed in Global Health as a field. In the sections that follow, we will use Robert’s disconcertment in the field as an analytical sensitivity to deconstruct three conflicting moments and we show how Global Health works with imaginaries of ‘impact’, constructs a particular kind of ‘local collaboration’, and prioritises practices of projectification and epistemic privileging.

### Engaging with the Global Health impact narrative

The ethnographic vignette above (see Table 1) shows that the certainty that Robert obtained from following fieldwork instructions, of collecting data for improvement, disappeared whilst interacting with the chairperson. The disconcertment within this moment arose after the chairperson confronted Robert with the irrelevance of the intervention to their village. This confrontation with the situated irrelevance of the intervention made Robert realise that – outside of the Global Health ‘impact narrative’ – he had little idea what it was that he was busy doing, or what he was meant to be productively engaging with. What stands out in this description is that Robert was sceptical about the necessity and value of his interventions from the onset, but he replaced these feelings of doubt with a belief that his engagements were meaningful as to be able to function in his role as Global Health researcher. Once the foundation of this ‘doing good’ belief was questioned by the chairperson, Robert’s role no longer felt as viable.

**Table 1:** First auto-ethnographic vignette

My initiation as a ‘global health researcher’ *in practice* was in 2016. In October 2016, I travelled to the capital of a country in the Global South and travelled onwards to a rural region to collect data on an intervention by a development organisation. At 24 years old, this was my first time to set foot on the African continent. After some days of accommodating to this new place, I was tasked with obtaining village chairpersons’ permission to conduct surveys in their respective local councils. Such interactions would roughly follow the same pattern: I would sit on the back of a *boda boda*, ^a^with– in my worn backpack – a notepad, informed consent forms, a pencil, and a bottle of water. I would pay the *boda* driver a day-rate that included gasoline costs and a compensation for their role as language interpreter.
On a regular day, we would drive over muddy roads searching for village chairpersons and as we arrived the alleged home of a chairperson, the driver would wander around the premises shouting ‘hello’ in a regional language. If the chairperson was home, I would usually ask the driver to explain the purpose of our visit. Commonly, we were invited to sit in the garden, in the shades of a mango, avocado, jackfruit, or papaya tree (the latter offering little shade), and the first order of business would be signing a guest book. After signing the book, I – through the driver’s translation – would start explaining that we were about to embark on a survey study in the chairperson’s constituency and that we would greatly appreciate it if the chairperson could offer their written support. In addition, we would ask the chairperson to draw up a map of the village, with a clear indication of household density and noting landmarks in the village. In most villages, this would be a seamless process and the chairpersons would have few questions or reservations.
It often felt, and looked, like it was standard procedure for the village chairpersons to have a researcher asking them for fieldwork permission. On the contrary, I would be quite uncomfortable and anticipated them to utter objections. I would worry, for instance, that they would criticise the lack of respondent compensation, or more importantly perhaps: that they would conclude that my study would not be of value to their village. But as we reached the end of our long list of villages from which to arrange permission, and taking the absence of objection as the presence of affirmation, I became increasingly convinced that I was in fact engaging in something meaningful.
When we visited one of the last villages on our list, the dynamics felt different. As before, the chairperson asked me to expand on the purpose of our visit. After I explained that we were collecting data on a development intervention, the chairperson asked several follow-up questions and I tried to clarify as much as possible. Finally, the driver conferred that the chairperson had asked: *“And when will you install the new water pump?”* (*fieldnotes).* I looked at the chairperson and at the *boda* driver, waiting for them to burst out laughing. But the chairperson was not joking. My stomach filled with cramp, and an intense feeling of bodily discomfort left me waiting for the chairperson to clarify their question; did they really think that I was here to install a water pump? My mind kept shifting between (a) how I would convince the chairperson that our study was really necessary and (b) whether our study would, in fact, be relevant at all. The chairperson calmly explained that they understood, of course, that water pumps were not a matter of my concern. But what was of my concern (i.e. studying the impact of an intervention) simply was not, at that time, in the best interest of his village. We would be allowed to conduct our study in the village, but the chairperson made it very clear that they would be doing us a favour, and not the other way around.

^a^The popular name for a motorcycle taxi.

The impact narrative, and its emphasis on interventions,^4^ is very much at the heart of Global Health. The narrative commonly develops as follows: “Look at this population, *their* health is poor and in urgent need of improvement. *We* need to intervene, and *our* intervention will improve *their* health. Here: these are *our* data in support of *our* intervention and these show that *their* health has indeed improved.” As shown in this fictive, but nonetheless accurate example, the impact narrative within Global Health follows a logic in which interventions are necessary to improve health locally, which are ultimately deemed to create better health globally. The ‘impact’ within this narrative is the *raison d’être* for Global Health: it is simply impossible to conceive of an unimpactful Global Health that does not aspire to improve health through intervention. Moreover, these interventions are usually brought in from outside the ‘environments’ in which they take place – which is also reflected in the use of the word impact.^5^ This produces a Global Health system that is directed at making impacts through intervention, and these impacts need to be measurable to account for the success of the intervention.^6^ This was also the exact reason why Robert was there in the first place; the household studies he was performing were meant to evaluate an intervention designed by a foreign development organisation to improve the health of *them*.

In practice, the Global Health impact narrative comes to surface at different levels. The narrative is present in mission statements of numerous Global Health faculties, non-governmental organisations, thinks tanks, and other entities. One of the more prestigious Global Health ‘schools’ writes for instance that it *“brings together dedicated experts from many disciplines to educate new generations of global health leaders and produce powerful ideas that improve the lives and health of people everywhere.”* Similarly, Global Health journals commonly explicate that they focus on improving health equity through impactful research. Zoomed out even further, and as described before, the UN’s third Sustainable Development Goal aims to *“ensure healthy lives and promote well-being for all at all ages.”* It is important to note that such statements about Global Health are not harmless, but define to a large extent how activities within that field are funded, organised, and governed.

A key problem with impact narratives is that they also fulfil a role in legitimising the interventions by Global Health actors. In practice, they are for instance also used to account for project expenditures, obtaining new funding, and to show the efficacy of promising interventions. Besides, academics in Global Health may use accounts of their ‘impact’ in appraisals of their performance. What this creates is a situation in which being ‘impactful’ likely goes at the expense of being reflexive. This is clearly visible in the vignette at the beginning of this section: Robert was tasked with evaluating a health system intervention and felt partly responsible for showing its impact. Alternatively, he might have invested in studying and reflecting on ways of making impact that would be more demand-driven. Such reflexivity may include questioning who defines and decides what makes Global Health interventions impactful, for whom, and to what extent such interventions produce unwanted effects. Furthermore, there is often a significant discrepancy between this global impact imaginary and local needs. Such discrepancies may be further augmented, or at least reproduced, by the fact that the interventionists come from elsewhere.^7^ Yet, the example also shows that the chairperson did not question Northern interventionism as such (as they still asked for a pump), but specifically did not support this extraneous intervention.

What becomes clear through the disconcertment in Robert’s encounter with the chairperson is precisely this conflict between the Global Health impact narrative and a practice that potentially does not fit within this wider narrative. The Global Health impact narrative is something Robert very much internalised through his training. Following the logic in that narrative, Robert may argue that his analysis of the intervention did result in knowledge about the efficacy of that intervention. Such knowledge can be used by the development organisation to expand their operations, but also to convince others of the success of their intervention. In addition, Robert was able to publish a scientific article about the intervention, which in theory allows other scholars to learn of the success of the intervention, but which also furthers his career. The problem is that the narrative itself can present a very powerful fiction that suggest that you are doing something meaningful, whilst the underlying uncomfortable – and more reflexive – question that remains is: what legitimises intervention in this specific situation? This is a particularly salient question given that the impact narrative as systemic aspect to Global Health generates and sustains a dependency in which Northern scholars are consequently the interventionist and Southern countries are places in which to intervene.

### Constructing the local Global Health collaborator

Even now, when putting the interaction with Joshua on paper (see Table 2), Robert still has conflicting feelings about his engagements with Joshua. This disconcertment thus did not present itself in a singular moment, but rather became more pronounced following the gradual regression of the intention to create a collaborative research project into a transactional and well-delineated one-off interaction: Joshua would arrange the institutional clearance and he would offer some advice on navigating national regulations on health research. In return, Joshua would be compensated for arranging the ethical clearance. The question that we think underlies this example is: why does the shift towards a transactional ‘collaborative’ arrangement produce feelings of remorse and disappointment in Robert as a Global Health researcher? And on a wider level: what brought about this regression?

**Table 2:** Second auto-ethnographic vignette

As with most Global Health research projects, this current project’s fiscal origin was the Global North. It was after the preliminary objectives were set that a process of engaging a ‘local collaborator’ started. My local collaborator was Joshua.
The first time I met Joshua *in person* was at the terrace of a university guest house in the Global South. We had made our appointment weeks before via e-mail, after I was referred to Joshua by a fellow researcher at a different Northern university. This academic colleague used to be Joshua’s PhD supervisor, but was also familiar with the development organisation whose activities we were now asked to evaluate. The colleague wrote that *“If you are looking for assistance in [city], knock at their doors, because these are well-trained people.” (e-mail correspondence August 2016)*. Joshua replied enthusiastically to my e-mail in which I described that I would visit the Southern country to make *“first contact”* for a pilot study and would be *“consulting the [university] ethical board” (e-mail correspondence September 2016)*.
Joshua arrived at our meeting in a worn Toyota sedan with one side-mirror hanging by an electrical cord, yet he was impeccably clothed. I would later write in my fieldnotes that I *“felt comfortable, because [Joshua] did not seem to notice my insecurities” (fieldnotes).* Joshua calmly discussed his previous research in which he studied local health systems, whilst we drank tea and ate toast with sunny side-up eggs. The timing of the new study, Joshua argued, was immaculate, as the government was seeking to implement a new local health system. This new system was supposed to prevent a dynamic *“where the performance of [health workers] drops as soon as the supervising NGO or implementation partner leaves,” (fieldnotes)* and provided sufficient cause for further qualitative research, Joshua argued. At the end of the first meeting, I asked Joshua about the procedures for obtaining ethical clearance. Joshua emphasised the necessity of moving through institutional review and did not foresee any issues if we anticipated about 300 US dollars of expenditures related to that procedure. My final notes of that meeting were: *“I do not want to put Joshua to work without having arranged a partnership agreement, be it informally.”* We agreed to discuss further in Vancouver, where we would visit the same conference in November.
Joshua and I met again on an autumn day in Vancouver, 14,027 km (8,715mi) away from our earlier encounter. It had only been two months since our first acquaintance, yet there was a stark difference in the nature and dynamics of that meeting. We sat down in the leather chairs of a café within the conference centre, while raindrops clouded our views on the harbour and what seemed to be an endless stream of departing hydroplanes. The insecurities that I felt during our first meeting had made way for feelings of excitement, and my dirt-stained clothes for a navy-blue suit. I had invited a senior colleague to join the meeting that I had so proudly arranged and was excited to finally discuss the substance of our collaborative research project.
Instead of discussing the substance of a collaborative research project, the meeting in Vancouver mainly revolved around financial arrangements. Joshua explained that there were three possibilities for collaboration: Joshua could (1) send an invoice for specific activities, (2) work on consultancy basis for a daily fee, or (3) become a co-investigator in the research project. The former two options, Joshua explained, would be relatively costly, while the third option would be more *“budget friendly”* (fieldnotes) but implied that Joshua would take part in project decision-making. In that discussion it was decided to start with the first option, with the possibility for a more extensive collaboration at a later stage should a more substantial budget be obtained. Several weeks later, Joshua sent me an invoice for 425 US dollars which covered Joshua’s work to arrange ethical clearance for the study. Despite earlier intentions, I would have only five more brief interactions with Joshua in the four years to follow – two of which were via e-mail. A final e-mail correspondence followed on the submission of a manuscript: *“Thanks Robert, all the best, J”*.

To understand what underlies Robert’s disconcertment in his interactions with Joshua, it is worth looking at what principles Global Health applies to collaborative research practices. What stands out in the Global Health literature that focusses on collaborative research, is a call for ‘fairer’ research, or even decolonisation of Global Health. These approaches have in common that they critique a Global Health in which researchers from the North practice ‘parachute’ or ‘parasitic’ research [30]: meaning that countries in the South are merely used for data collection, and academics from the South are used only to provide access to the field or for legitimation purposes. The alternatives that are presented focus on collaborative practices that are ‘fairer’, more equitable, that are based on demands and priorities of communities in the country of study, led by researchers from the country of study, and in which there is no a priori superiority of Northern knowledge, logic, and method [8, 31, 32].

From the outside, Robert’s transactional arrangement with Joshua looks like a direct antipode of what contemporary Global Health literature envisions as a fair research collaboration. This stark difference may partly explain the disappointment that Robert experienced afterwards. By identifying himself (and in being identified by others) as a Global Health researcher, Robert positions himself – at least partly – in a wider ideological frame in which this transactional type of collaboration is deemed ‘wrong’ and potentially harmful. Robert thus knew that he was complicit in a practice that the Global Health field denounces. More pragmatically, Robert’s disappointment stems from his anticipation that with Joshua’s participation in the study, he could learn from Joshua’s experience doing fieldwork and extensive knowledge on local health systems. Instead, Robert was involved in enrolling Joshua as a facilitator and Robert worked as a relatively solitary and isolated academic ‘in the wild’, without any experience doing fieldwork, and in an environment of which he did not understand most of the languages and customs. This was particularly frustrating given that Joshua had worked on similar interventions in his country for several years and was in close contact with health authorities in the region where Robert conducted the epidemiological study.^8^ What played a role in this type of engagement with Joshua is that there was no explicit budget available for local collaboration, and Robert experienced little leeway being an early-career researcher. This made that Robert was reluctant to discuss other (non-remunerated) ways of engaging Joshua, as he felt that this was not in accordance with standards for fair collaboration.

This example of Robert’s transactional arrangement with Joshua is emblematic of how a substantial part of Global Health works. Historically, the field has committed to epistemic practices where data are collected in the ‘fields’ of the Global South and subsequently processed, analysed, and translated into a peer-reviewed scientific publication or project report in the Global North [33]. The early 1990s marks the start of a movement within Global Health that seeks to counter this dynamic and that calls for more Global Health research by the Global South, for the Global South.^9^ In turn, some Global Health research funders and journals began stipulating requirements to facilitate this shift. Some journals for instance implemented a compulsory ‘reflexivity statement’ policy, or requested compliance with an extended interpretation of authorship criteria.^10^ Moreover, countries in the Global South increasingly require foreign Global Health scholars to apply for a research permit, which demands an affiliation with an academic institution in that country. The ‘local collaborator’ as construct, of which Joshua is an example, is as much a way to abide to these policies as it is a strategy of circumventing them. This circumvention is the result of an academic Global Health system in which local collaboration is rarely financially supported and often considered as a means to an end; the ‘end’ in Robert’s case being the task to scientifically reflect on the performance of a health system intervention developed by a Northern organisation. This systemic aspect to Global Health practice is persistent and entangled with dynamics of accountability, epistemic privileging, and personal career prospects.

Considering the collaboration with Joshua we may argue that this interaction was relatively ‘fair’^11^ in its transactional nature. The project clearly benefitted from Joshua’s experience with the health research system in his country, including its logistics and requirements. Joshua was, in that capacity, crucial for the success of the study. For Joshua, on the other hand, this was one of many transactional arrangements that he was involved in, and he explained that this is simply part of his job and a key component of his monthly income.^12^ At the same time, and as becomes clear through Robert’s disconcertment that ensued the collaboration, the transactional interaction worked around Joshua both as important source of knowledge and *knower*. By working with Joshua as ‘local collaborator’, Robert did not contribute to the development and maintenance of national knowledge infrastructures. Instead, he contributed to and maintained part of the Global Health system that values professional ‘local collaborators’ over other, more productive types of collaboration.

### Producing Global Health knowledge

“We need to make sure that we have all data on everything, because the funder but also our research integrity code requires us to have everything stored at secured servers, etcetera, etcetera. So, we need to make sure that all data is transparent and securely stored.”

George, Rotterdam, 2020

The brief excerpt above and the vignette in Table 3 can be read as an observation of any arbitrary research project meeting. The discussions about planning, deliverables, and funding, a project actor that negotiates with another consortium member, and a disconcerted PhD candidate who needs to coordinate it all: these do not seem like dynamics unique to Global Health research. What can be seen as unique to Global Health research are the tensions that may arise from the friction between the normative ambition of Global Health research (i.e. to contribute to better health), a projectified research practice that is mostly attentive to deliverables, publications, funding, and overall accountability, and fiscal and administrative dependency of Southern Global Health actors on Northern organisations. In the project at hand, we explicitly aimed for a ‘locally-led’ and ‘demand-driven’ practice, yet such an approach requires a flexibility that is not inherent to the logic of projectified research. To Robert personally, the disconcertment of these conflicting, or incongruous logics, lies in the eventual prioritisation of ‘accountability’ over other motives of the project and the fact that he, as a PhD candidate, would be the one enacting that accountability by constantly monitoring and evaluating the practices of Robert’s more senior colleagues in two Southern countries.

**Table 3:** Third auto-ethnographic vignette

The meeting takes place in a small conference room on the 17th storey of a university building and the sunlight impairs the view on the overhead screen. I am trying to take a panorama photo of the city skyline, whilst my colleagues from the Global South attempt to join the wireless guest network. This is the first day of a two-day ‘end-of-project’ meeting, which was actually scheduled for June but was postponed for four months due to delays in the visa applications of our colleagues from abroad. Our formal agenda is to discuss the ‘lessons learned’ of the project and to decide on any potential scientific outputs.
Our final item just before lunch is the sharing of data. A project member explains that we are now gathering all data in a *“secured server”*^a^ as per university and European data protection regulations. Peter, a Southern professor, responds that it is entirely unclear for their team what should be uploaded where: *“(…) what data are we talking about?”.* The professor, who only became involved later on in the project, subsequently says that they might have misunderstood part of the data collection and asks whether we could explain that more clearly. It is decided that I will make a data checklist that clearly shows what data should have been collected and what needs to be uploaded to the digital storage. Tomorrow’s agenda will therefore allot some time to discuss data practices, beside the scheduled *“project deliverables”*, *“planned publications”*, and *“next funding”*.
The second day of our project meeting starts with a discussion about one of the research methods we further developed. The project proposal stipulated that we would use this research method to conduct ten case-studies in all three countries, but thus far this activity has commenced in one of the project countries only. Peter explains that the interviews have not yet started in his country and that he is opposed to doing interviews *“across the country”*, as travel is *“very expensive and logistically too intensive”* (fieldnotes). One project member is not quite satisfied with this answer and explains that it is important that the process is completed, certainly as we promised the funder that we would do it *and* because we need the data for our analysis. After a short deliberation, Peter concedes that *“they are happy to do it”*, but it requires more budget and if that is to be made available, November will be *“activity time”* (fieldnotes) in his country. It is decided that the number of interviews will be reduced and that interviews will take place over telephone.
I realise that I will be the one to check and monitor whether the teams in the project countries conduct all activities as planned and promised. In practice, this means sending e-mail reminders every week and texting my senior colleagues in these countries until we receive a full report that is to our satisfaction. This is precisely what I have done throughout the past two years and which gave me a constant feeling of policing and belittling them – certainly as they are generally more advanced in their academic career and experience than I am.

^a^Which was in fact just a simple Google Drive folder, because that was more accessible to colleagues in the Southern countries.

An important question is why projectified research, and the accountability schemes that come with it, threaten a more reflexive research approach, and to what extent this is a more systemic aspect of Global Health. We argue that in our project, the prioritisation of accountability is a symptom of a wider academic culture within Global Health (and beyond) that regards scientific publications higher than, for instance, use of research evidence in policymaking processes of Southern countries. Our project explicitly set out to both study *and* improve the utilisation of knowledge in the project countries, but the former would constantly challenge the latter. In the project meeting, this came to the front when a project member realises that, for the purpose of writing a scientific paper about a specific method, insufficient data had been collected. Subsequently all kind of changes are applied to make sure that (i) there are sufficient data that are suitable for writing scientific publications and (ii) a minimal level of methodological quality is met. These changes include the relaxation of certain methodological criteria (telephone interviews instead of face-to-face, less interviews and cases), but also the mobilisation of actors known for their meticulous accounting practices – such as ‘the funder’ and the ‘research integrity code’. At the same time, Robert is tasked with more intensively monitoring the data collection practices of the project countries on a weekly basis. Apart from Robert’s experience that this created a reversal in hierarchy, it also led to a complicated dependency: the teams in the project countries were now expected to completely abide to the project planning if they wanted to receive the final instalment. This is particularly salient given that, compared to the relative security Robert derives from his appointment at a Dutch university, research organisations in the Global South are more likely to rely on project funding for their sustaining.^13^

A critical analysis of the project presented above could argue from the outside that it does not abide to Global Health’s normative agenda at all: how else could it be that the impact narrative comes to be challenged by logics of accountability? We argue instead that the example here demonstrates that there are commonly dissociable agendas at play in Global Health research projects, and that – despite honest and good intentions – these agendas can conflict with the wider normative agenda of ‘doing good’ in Global Health. Robert, for instance, wants to finalise his PhD as this gives him entrance to an academic career.^14^ One of the project member’s agendas is to further develop and validate a scientific method that they developed and held dearly. The research funder wants to fund ‘impactful’^15^ projects and the financial controllers of the university would like to close the cashier within the formal project period. These agendas have different (epistemic) requirements, but they have in common that they do not facilitate the production of knowledge that is not necessarily generalisable, that is not readily appropriate for scientific publication, and which may be of use only to actors within the environment where that knowledge was produced.

Our Global Health research project is not alone in sometimes privileging academic knowledge production.^16^ We argue that this is indeed what most Global Health research projects do, and which also allows the projects to have measurable ‘impacts’ within the project time frames. While it is not our intention to offer a universal epistemological taxonomy of Global Health, we do argue that a substantial part of Global Health research adheres to a positivist epistemology. Following this epistemology, sophisticated research designs are applied to distil ‘data’ from Global Health’s ‘fields’ with as little distortion as possible. The researchers themselves are deemed (and ought) to be objective and purely work as blinded operators of their research designs and software packages. Only when these procedures fulfil the highest norms of validity and precision, objective truths can be ‘found’. These truths, for instance about the performance of a Global Health intervention, are subsequently published in the scientific literature and the assumption is that others may then use the same intervention in a different Global Health setting. These systemic aspects can be summarised with terms like replication and (empirical) generalisability and they are not reserved for positivist Global Health research practices only. Constructivist research practices within Global Health equally assume a theoretical generalisability to some extent. Such studies work through an inductive logic that argues that ‘patterns’ or ‘mechanisms’ can be distilled from studying empirical phenomena, and that these understandings – albeit constructions – are supposed to have some validity at a different place as well. Which still leaves Global Health prone to parachutic research practices.

## Discussion and conclusion

In the introduction of this paper, we positioned Robert’s unease regarding ‘good’ Global Health scholarship within a wider dispute over Global Health’s intentions and achievements. Particularly, we argued that we may understand this normative dispute better by analysing moments of disconcertment that occurred in Robert’s work as Global Health scholar. By collectively analysing three auto-ethnographic vignettes from Robert’s fieldwork, we sought to interrogate Global Health’s normative agendas and offer a personalised, situated, and reflexive account of how such agendas work out in practice. The analysis, and the conclusions we present, are very much situated in Robert’s personal disconcertment. Nevertheless, and looking at the literature that critiques Global Health, we argue that Robert’s disconcertment provides insight into dynamics that are recognisable to other actors within Global Health. Therefore, we want to translate the analysis of this paper into a set of areas and elements to be aware of when working in Global Health. That is not to say that Robert’s disconcertment is universally true or generalisable: instead, it allowed us to construct insight into more systemic characteristics of Global Health. To be precise, our analysis shows three overarching ‘systemic’ imperatives to being a ‘good’^17^ Global Health scholar:

I. Thou shall have impact, in academia and your ‘fields’.
II. Thou shall collaborate, fairly.
III. Thou shall stick to the project plan, at least on paper.

In these final paragraphs of our paper, we seek to do four things. First, we will position the three imperatives presented above in the wider (critical) literature on Global Health. In particular, we explore how our identification of these three imperatives through an analysis of disconcertment adds to the literature on ‘what is wrong’ with Global Health. Second, we will expand on why disconcertment in Global Health practice should not be disarmed, but how instead its momentum can be used to construct more productive realities. Third, we aim to reflect on the limitations that are inherent to our approach – the most prominent being that this is yet another Northern account of Global Health. We conclude the section with two suggestions for further action and study.

The three imperatives presented in our discussion are incomplete, and obviously (somewhat) caricaturised in their wording. Yet still they echo earlier observations in the wider Global Health literature. Holst [34], analysing a plethora of Global Health definitions, for instance notes that the field demands a focus on interventions and their impact, which distracts from integrating such interferences from outside with national policies. Gautier et al. [6] highlight how ‘partnerships’ in Global Health have led to more collaboration, and higher access of Southern academic organisations to Global Health as a field, but such partnerships structurally reify and augment existing inequalities and unproductive dependencies. ‘Our’ third imperative, which focusses on dynamics of projectification and accounting, has also been observed in Global Health before [35–37]. We chose to describe these observations as imperatives here to emphasise that they are commanding and persistent in nature and being a ‘good’ Global Health scholar means that you have to work with them.

Saliently, the imperatives are difficult to combine as they require different activities, methodologies, and procedures. Practices of accounting, for instance, benefit from a clear planning, hierarchical structure, and strict measurement of deliverables, whereas fair collaboration necessitates flexibility, adaptability, and reciprocity. The implication of this divergence is that actors within Global Health constantly need to navigate through conflicting goals and accountability networks. As we have shown, it is precisely such conflicts which may produce (and that can be explicated through) disconcertment – which makes such moments the metaphorical canary in the coal mine and introduces the question: how can we use such disconcerting moments productively?

Inspired by the work of Haraway [38], we propose that moments of disconcertment can be made productive by *staying with* them, rather than disarming them. The difference between the two is important and so we will explain it here using the first vignette as example. In that vignette, a chairperson argues that a different intervention is of more relevance to his village. A strategy directed at disarming this disconcertment provides Robert with four choices, or a variation thereof: (a) ignore the chairperson and continue his job, (b) take over the chairperson’s suggestion, (c) convince the chairperson that the intervention *is* relevant, or (d) not intervene. But as we have shown in our paper, neither of these options would be satisfactory as they do not question what underlies the disconcertment but reduce it into a mere problem that can be prevented, ignored, or resolved. We contend here that staying with the disconcertment requires different strategies, at different levels. In the example presented, that could mean that the disconcertment works as a reflex to Robert, which brings him to discuss his conflicting feelings with the chairperson. Similarly, Global Health educational programmes may teach students not to disarm disconcertment, but to openly discuss it instead. On a project level, staying with disconcertment may require building new collaborations, or the creation of more flexible spaces within the project that allow for deviation of protocol and planning [39]. What this shows is that disconcertment is not something that needs to be resolved, but a reflexive diagnostic which can interrogate existing assumptions, patterns, and roles.

Our analysis presented several limitations, and they culminate into the following question: who are *we* to claim that *we now know* what is wrong with Global Health? The analysis of this paper started with Robert’s initiation into Global Health as a loosely demarcated field and the disconcertment that arose in his practices. By collectively analysing Robert’s personal and often embodied experiences we sought to ‘zoom out’ and place them in the wider dynamics of academia in general, and Global Health specifically. It is important to stress once more that this analysis must not be seen independent of our own positions and roles. Our analysis does not provide generalisable truths and staying true to the disconcertment of other scholars and voices will yield different analyses and likely identifies other imperatives than those described by us. It is not unreasonable to assume that for scholars seeking to decolonise Global Health, our approach does not reach far enough (at all). We also do not seek to obfuscate our complicities in Global Health and confirm that our analysis is very much inscribed by who we, as white, male, Western scholars at various stages of our academic careers, are. Yet, we also think that it in that capacity it is our responsibility to contemplate how we can create different, more reflexive, less restrictive, and pluralistic global healths – which is precisely the endeavour that this paper seeks to contribute to.

Having reached the end of this paper, we want to point out two opportunities for further action and research. First, while the recent Global Health literature has produced numerous critical commentaries, discussion pieces, and editorials that highlight problematic aspects in Global Health, there are still few detailed (auto)ethnographic accounts of how these problematic aspects worked out and were experienced in practice, both by scholars from the South and North. Most Global Health journals are structured in such a way that classical ‘ethnographies’ do not fit, and as such they end up in disciplinary journals where most of the ‘Global Health audience’ does not reside. Institutionally facilitating lengthier and less rigidly structured papers seems like an important and straightforward first step here. Second, the imperatives we have described in this paper are not unique to Global Health, but some features may be more pronounced there. Most prominent herein is the organisation of consortia where Northern academic performance schemes and financial structures impair equitable collaboration. It is therefore that we deem it important to bring such imperatives to the front and to scrutinise them. Moments of disconcertment can play a key role in this. It is safe to say that if Robert would have stayed with his disconcertment, and discussed it with the actors in those moments, this paper would not have existed in its current format. Besides, he may have never been asked to install a water pump, but to do something else instead.

## Data Availability

All data generated or analysed during this study are included in this published article.

## Abbreviations

LMIC: Low- and middle-income country
NGO: Non-governmental organisation
STS: Science and Technology Studies
UN: United Nations

## Footnotes

1 This ‘Global North’ is global nor Northern, and the same holds true for ‘Global South’. These terms are used in this manuscript as problematic and coarse descriptors of a select group of countries. The ‘North’ represents countries in which renowned ‘Global Health’ organisations are based, but in which those organisations generally do not conduct their activities. In practice, this results in a set of ‘high income’, likely (former) colonising, and often Anglophone countries. Our main rationales for using the term here is to adhere to language used by Global Health scholars and to ensure the anonymity of the actors involved. See also Haug et al. [40].

2 During a graduate school seminar, Robert was blamed by a fellow PhD candidate for practicing ‘white saviorism’ when confessing such uncertainties, which was precisely the opposite of what he meant: he intended to critically inspect his own intentions and complicities when ‘doing good’. Which is what we also aim to do here.

3 This is a reference to the title of a collection of papers in *The Lancet Global Health*: https://www.thelancet.com/what-is-wrong-with-global-health.

4 The word intervention is commonplace in Global Health and usually connotes a practice of interference in specific settings and environments with the idea that this interference will improve the health or well-being of actors in those environments.

5 See for an extensive discussion for instance Kok & Schuit [41]. They parallel the concept of ‘impact’ (and its usage) to a cannonball that is assumed to transfer its momentum through collision when shot with sufficient force and after meticulous targeting. They argue that the intensity of collision lies not in the force of the metaphorical cannonball (e.g. an intervention), but in how actors work with that intervention in practice.

6 See Greenhalgh et al. [42] and Penfield et al. [43] who make a similar claim.

7 The notion of ‘elsewhere’ is at the core of global health, as global health practitioners generally work in countries other than their own, and the field aims to transfer methods, interventions, and knowledge between places. Bibliographic studies show that this ‘elsewhere’ quite persistently refers to a small set of countries only [7, 44].

8 This Robert only found out later, after an interviewee noticed that Joshua’s name was on our documents.

9 For an extensive discussion, see Edejer [45] or Lansang & Dennis [46].

10 Both the mentioned ‘reflexivity statement’ [47] and authorship criteria [48] are relatively recent developments, which were preceded by e.g. mandatory LMIC co-authorships.

11 In the sense of “free from bias, fraud, or injustice; equitable; legitimate, valid, sound” [49].

12 Borst et al. [36] similarly show that such dependencies can be productive and nonetheless oppose certain norms, for instance about what is seen as ‘good’ global health research practice.

13 See Borst et al. [36] for a comprehensive analysis of the dynamics of projectification in Global Health.

14 In most Dutch academic practices, completing a PhD requires writing a thesis that is based on at least four scientific publications – some of which also need to be accepted and published by a scientific journal.

15 This is a reference to our first argument on Global Health’s ‘impact narrative’, which – as we show – may produce less-reflexive practices.

16 Similarly, Boaz et al. [39] and Heney & Poleykett [18] show how current institutional arrangements in academia privilege knowledge production processes directed at producing generalizable ‘truths’ that benefit the reproduction of academic norms and identities.

17 This is precisely to connote that to be a productive Global Health scholar, who is academically prolific and who ties into Global Health networks, means obeying these problematic imperatives.
